# Improving the Management of Patients with Hearing Loss by the Implementation of an NGS Panel in Clinical Practice

**DOI:** 10.3390/genes11121467

**Published:** 2020-12-07

**Authors:** Gema García-García, Alba Berzal-Serrano, Piedad García-Díaz, Rebeca Villanova-Aparisi, Sara Juárez-Rodríguez, Carlos de Paula-Vernetta, Laura Cavallé-Garrido, Teresa Jaijo, Miguel Armengot-Carceller, José M Millán, Elena Aller

**Affiliations:** 1 Group of Molecular, Cellular and Genomic Biomedicine, IIS-La Fe, 46026 Valencia, Spain; berzalserrano@gmail.com (A.B.-S.); rebeca.va95@gmail.com (R.V.-A.); sarajuarezrodriguez@gmail.com (S.J.-R.); cavalle_lau@gva.es (L.C.-G.); jaijo_ter@gva.es (T.J.); miguel.armengot@uv.es (M.A.-C.); millan_jos@gva.es (J.M.M.); aller_ele@gva.es (E.A.); 2 CIBER of Rare Diseases (CIBERER), 28029 Madrid, Spain; 3 ENT Department, Polytechnic and University Hospital La Fe, 46026 Valencia, Spain; piedadgardiaz@gmail.com (P.G.-D.); depaula_car@gva.es (C.d.P.-V.); 4 Surgery Department, University of Valencia, 46026 Valencia, Spain; 5 Units of Genetics, Polytechnic and University Hospital La Fe, 46026 Valencia, Spain; 6 CIBER of Respiratory Diseases (CIBERES), 28029 Madrid, Spain

**Keywords:** hearing loss, next-generation sequencing, genetics, molecular analysis, clinical evaluation

## Abstract

A cohort of 128 patients from 118 families diagnosed with non-syndromic or syndromic hearing loss (HL) underwent an exhaustive clinical evaluation. Molecular analysis was performed using targeted next-generation sequencing (NGS) with a custom panel that included 59 genes associated with non-syndromic HL or syndromic HL. Variants were prioritized according to the minimum allele frequency and classified according to the American College of Medical Genetics and Genomics guidelines. Variant(s) responsible for the disease were detected in a 40% of families including autosomal recessive (AR), autosomal dominant (AD) and X-linked patterns of inheritance. We identified pathogenic or likely pathogenic variants in 26 different genes, 15 with AR inheritance pattern, 9 with AD and 2 that are X-linked. Fourteen of the found variants are novel. This study highlights the clinical utility of targeted NGS for sensorineural hearing loss. The optimal panel for HL must be designed according to the spectrum of the most represented genes in a given population and the laboratory capabilities considering the pressure on healthcare.

## 1. Introduction

Hearing loss (HL) is the most common sensory deficit in humans [1]. According to data from the World Health Organization, it is estimated that more than 5% of the world’s population suffers from this disease, that is, around 360 million people. 

HL can be classified as conductive, sensorineural or mixed (a combination of both); acquired or hereditary; prelingual or postlingual; and non-syndromic (NSHL) or syndromic, as a part of a more complex phenotype, that account up to 30% of HL cases [2].

HL is one of the most common birth defects, with an incidence of 1–2 per 1000 newborns and growing as age increases, reaching more than 300 per 1000 in those over 75 years of age. This high incidence is due to both environmental and genetic factors. The genetic contribution to newborn HL has been reported to be 50–60% depending of the study and the population [3,4].

As the rate of acquired hearing loss secondary to environmental causes decreases, the significance of genetic factors that lead to deafness increases [5]. To date, over 120 genes have been associated with NSHL (Hereditary Hearing Loss Homepage: https://hereditaryhearingloss.org/), and over 400 syndromes have been associated with hearing impairment [6]. These genes encode proteins of a very diverse nature and are involved in different pathways, such as mechanotransduction, ear structures, ion homeostasis, etc.

Genetic confirmation of hearing loss is essential to the provision of genetic counseling, to ascertain the risk of recurrence and, in some cases, to determine the prognosis and select the best rehabilitation options. Furthermore, although the utility of molecular diagnosis is still limited for therapeutic approaches, a growing number of gene-based strategies to treat HL have been carried out in recent years at preclinical stages [7].

In the last decade, next generation sequencing (NGS), including custom targeted panels and whole exome sequencing, has revolutionized the genetic screening of disorders with high genetic and allelic heterogeneity, such as hearing loss, allowing hundreds of genes in several patients to be screened simultaneously in a short time and in a cost-effective manner.

In this study, we assess the efficacy of a home-designed panel for hearing loss in the Genetics Department of a tertiary university hospital.

## 2. Materials and Methods

### 2.1. Patients and Samples

A total of 128 patients from 118 families diagnosed with non-syndromic or syndromic HL were included in our study. Most patients were of Spanish origin, except for three patients that came from Eastern Europe, two patients that were from Maghreb, two patients that were of sub-Saharan origin and one patient that was from East Asia. Patients were recruited from September 2017 to December 2019. Most patients presented with non-syndromic hearing loss, but we also received for screening four patients with Usher syndrome (USH), two with Waardenburg syndrome (WS) and two patients with branchio-oto-renal syndrome (BOR). Patients were enrolled through the Department of Otolaryngology of the University Hospital La Fe, according to standard assistance procedures. Comprehensive clinical evaluations, imaging examination, pure-tone audiograms, auditory brainstem response and other relevant medical information were collected for the probands to characterize the type and severity of HL. All recruited patients presented sensorineural or mixed HL. Hearing loss severity was established as mild (between >25 and ≤40 dB), moderate (between >40 and ≤70 dB) or severe/profound (>70 dB).

Written informed consent was obtained from all participants or their legal guardians. This study was approved by the Hospital La Fe Ethics Committee in agreement with the Declaration of Helsinki (REV03/5/2014).

Genomic DNA (gDNA) from the patients and relatives was obtained and purified using the automated DNA extractor QIAsymphony (QIAGEN, Hombrechtikon, Switzerland). The concentration of the resulting DNA samples was determined with Nanodrop and Qubit fluorometer (Thermo Fisher Scientific, Waltham, MA, USA)

### 2.2. Panel Design

We designed an NGS panel for the analysis of hereditary hearing loss using the SureDesign tool (Agilent Technologies, Santa Clara, CA, USA). The genes that were included in this panel were selected according to the prevalence reported in different studies [1,8,9,10] choosing those with the highest prevalence. Finally, the panel included the coding regions and flanking intronic regions (+/–25 bp) of 59 genes, 35 of them associated with non-syndromic HL, and 24 genes associated with syndromic HL (Table 1). The panel also included five deep intronic regions of the *USH2A* gene [11,12,13]. 

We tried to include some extra probes for the regions of *ESPN*, *OTOA* and *STRC* genes showing high homology with their pseudogenes, in addition to the default probes generated by the *SureDesign* software (Agilent Technologies, Santa Clara, CA, USA). Three extra probes were designed and included for *ESPN* (chr1:6500314-6500500, chr1:6500686-6500868, chr1:6505724-6505995) and seven for *OTOA* (chr16:21742158-21742251, chr16:21752042-21752229, chr16:21756202-21756357, chr16:21763256-21763398, chr16:21763690-21763826, chr16:21768403-21768598, chr16:21771791-21772050). However, bioinformatic tools failed to design extra probes for *STRC*, due to the fact that *STRC* is 99.6% identical to its pseudogene (*pSTRC*).

### 2.3. Library Preparation and Sequencing

The library preparation was carried out according to the Bravo NGS SureSelectQXT Automated Target Enrichment protocol (Agilent Technologies, Santa Clara, CA, USA) for Illumina Multiplexed Sequencing. Sequencing analysis was performed sequentially in batches of 16 patients. The libraries were sequenced on a MiSeq instrument with a MiSeq v2 300 cycle reagent kit (Illumina, San Diego, CA, USA). 

### 2.4. Data Analysis

The resulting sequencing data were analyzed with the Alissa software tool (Agilent Technologies, Santa Clara, CA, USA) in regard to the human assembly GRCh37/hg19. This software performs the alignment, variant calling and annotation of the variants. The annotated variants were filtered according to a minor allele frequency (MAF) value ≤ 0.02 (the frequency of the variants was explored in the Exome Aggregation Consortium (ExAC) database, genomeAD (https://gnomad.broadinstitute.org/) and 1000 genomes (https://www.internationalgenome.org/). To classify the variants, we also took into account their annotation in the dbSNP (www.ncbi.nlm.nih.gov/SNP/), their description in ClinVar (https://www.ncbi.nlm.nih.gov/clinvar/), Varsome (https://Varsome.com/), HGMD (http://www.hgmd.cf.ac.uk/), LOVD (https://www.lovd.nl/) and Deafness Variation Database **(**http://deafnessvariationdatabase.org/) and the variant type. Novel missense variants were evaluated with the predictors included in the Varsome website and Alissa software: *BayesDel_addAF, DANN, DEOGEN2, EIGEN, FATHMM-MKL, M-CAP, MVP, MutationAssessor, MutationTaster, REVEL* and *SIFT*. 

To predict the potential effect of the variants on the splicing, we used the bioinformatic tools *MaxEnt* and *Splice AI*.

Sanger sequencing (BigDye Terminator kit v1.1, Applied Biosystems, Carlsbad, CA, USA) was carried out to validate the pathogenic and likely pathogenic point variants and to perform segregation analysis when patients’ relatives were available.

To detect copy number variations (CNVs), we carried out an analysis using the DECoN v1.0.2 program [14], which is a tool that detects variants in copy number from aligned sequences based on the number of reads for each position. The CNVs obtained by this program were checked using the multiplex ligation-dependent probe amplification technique (MLPA): *OTOA* + *STRC* (P461 salsa) (MRC-Holland, Amsterdam, The Netherlands). Deletions previously described to affect the DFNB1 locus were confirmed by multiplex PCR [15]. These MLPA reagents were also performed in patients with only one pathogenic variant detected in a gene with (autosomal recessive) AR inheritance. 

## 3. Results

We aimed to obtain a median read depth greater than 100×. Coverages obtained were around 150×–200×, and 98% of analyzable target regions were covered by at least 20 reads. However, some regions of 3 genes with homologous pseudogenes (*ESPN, OTOA* and especially *STRC*) were not well covered. These regions are detailed in Table S1.

We detected the variant(s) responsible for the disease in 47 out of 118 families (40%), 27 with an AR inheritance pattern, 18 with AD and 2 with an X-linked pattern (Table 2). Detailed clinical data from the diagnosed patients are shown in Table 2.

**Figure 1:**
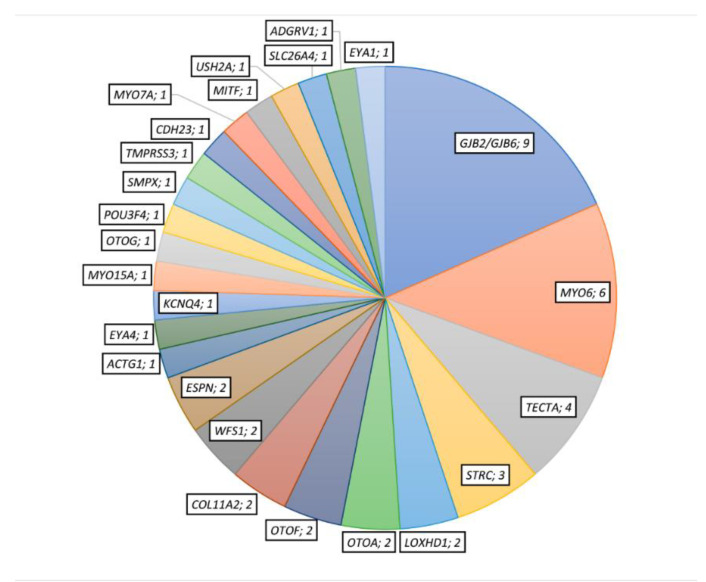
Number of diagnosed patients with putative disease-responsible variants in each represented gene.

We identified candidate variants in 26 different genes, 15 with AR inheritance pattern, 9 with AD and 2 with an X-linked pattern (Figure 1). Among the 54 different candidate variants detected, 24 were missense, 7 frameshift, 11 nonsense, 2 inframe ins/del, 3 CNVs and 7 affected to the splice-site. Fourteen out of 54 variants were novel (Table 2 and Table 3).

### 3.1. Autosomal Recessive HL

Twenty-nine cases belonging to 27 families carried biallelic pathogenic or likely pathogenic variants associated with an autosomal recessive pattern of inheritance (Table 2A).

**Figure 2:**
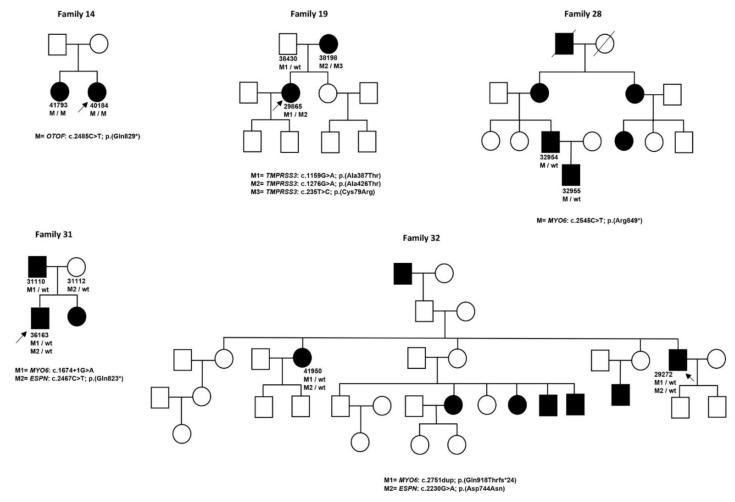

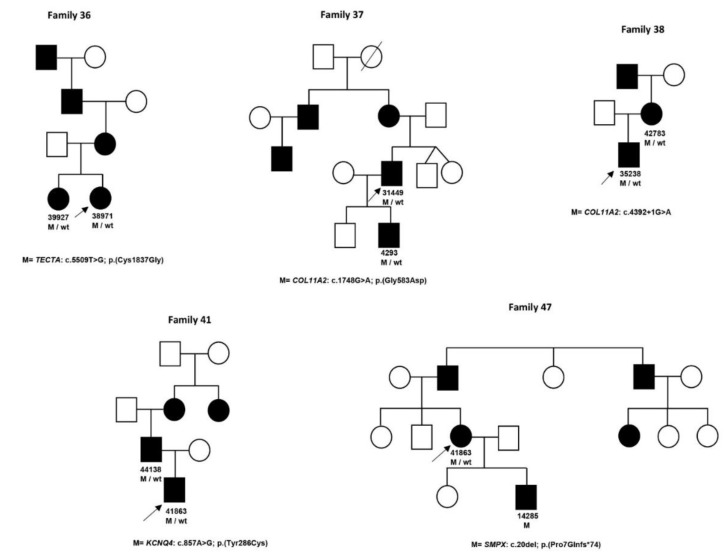
Pedigrees of the families and segregation analysis of the detected pathogenic or likely pathogenic variants. Arrows indicate the proband case, M indicates the pathogenic or likely pathogenic variant and wt indicates wild type sequence.

Twenty-six cases presented with NSHL. These were linked to *GJB2/GJB6* (DFNB1) (nine cases), *STRC* (three cases), *OTOF* (three cases belonging to two families), *LOXHD1* (two cases), *OTOA* (two cases), *TMPRSS3* (two cases belonging to one family) and one case in the *MYO15A, SLC26A4, OTOG, TECTA* and *MYO7A* genes (Figure 1). Family trees for families with more than one affected patient are displayed in Figure 2.

The remaining three solved cases suffered from Usher syndrome due to putative pathogenic variants in *ADGRV1, CDH23* and *USH2A*, one family for each gene.

The most prevalent variants found were c.35del (*GJB2*) and del (*GJB6*-D13S1830), both affecting DFNB1 locus, followed by the complete deletion of the *STRC* gene. In all cases, the deletion of *STRC* was associated with mild to moderate postlingual hearing loss.

Five of the detected pathogenic variants were novel. Four of them produced a premature stop codon: three frameshift variants (c.3419dup/p.(Leu1140Phefs *5) in *LOXHD1*, c.877C > T/p.(Gln293 *) in *OTOA* and c.2140dup/p.(Ser714Lysfs *22) in *OTOG*) and one nonsense variant (c.310G > T/p.(Glu104 *) in *CDH23*). The only novel missense variant detected was c.235T > C/p.(Cys79Arg) in *TMPRSS3*.

### 3.2. Autosomal Dominant HL

We identified variants responsible for the disease associated with an autosomal dominant pattern of inheritance in 25 patients belonging to 18 families (Table 2B). 

Twenty-four of these patients had been referred as non-syndromic HL. Nine patients belonging to six families presented variants in *MYO6*, four patients from three families in *TECTA*, four patients from two families in *COL11A2*, two patients from two families in *WFS1* and two patients from the same family in *KCNQ4*; pathogenic variants in *ACTG1* and *EYA4* were detected in one patient each (Table 2B and Figure 2).

One of the families linked to *COL11A2* (family 38) was found to present the pathogenic variant c.4392 + 1G > A, previously described by Brunner et al. (1994) [48] as associated with Stickler syndrome without eye affectation. This family was clinically re-evaluated and re-classified as Stickler syndrome.

Additionally, we also detected pathogenic variants in two families with syndromic hearing loss. We found the variants responsible for the disease in one patient diagnosed with Waardenburg syndrome, presenting the variant responsible for the disease in *MITF*, and one patient diagnosed with BOR syndrome was found to present with the pathogenic variant in *EYA1.*

No prevalent pathogenic variants associated with an autosomal dominant (AD) pattern of inheritance was detected. Seven of the AD pathogenic variants identified in the present study were novel. One novel stop codon (c.1666C > T/p.(Arg556 *)) was detected in *MYO6.* Two splicing variants, none previously described, were detected; one of them was located at a canonical site (c.1674 + 1G > A in *MYO6*), and the other was located at c.1224-9del in *MYO6*. Furthermore, an in-frame novel duplication was found in *WFS1*, c.1463_1474dup/p.(Val491_Pro492insLeuIleThrVal) and two missenses variants in *COL11A*2 (c.1748G > A/p.(Gly583Asp)) and *MYO6* (c.494T > G/p.(Leu165Arg)) (Table 3). 

**Figure 3:**
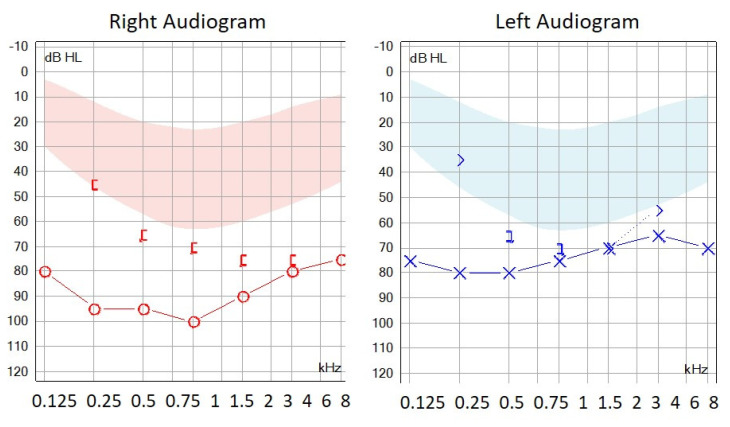
Audiogram performed in patient 40431 harboring the c.1463_1474dup/p. (Val491_Pro492insLeuIleThrVal) variant in the *WFS1* gene.

The audiogram of patient 40431, harboring the c.1463_1474dup/p.(Val491_Pro492insLeuIleThrVal) variant, showed a characteristic profile with severe threshold increases for low-frequency tones (Figure 3).

### 3.3. X-Linked HL

Variants responsible for the disease associated with an X-linked pattern of inheritance were found in three cases belonging to two families (Table 2C). One case presented a novel missense variant in *POU3F4* (recessive X-linked) and the other two cases were a boy and his mother, both carrying a novel frameshift variant in *SMPX* (dominant X-linked) (Figure 2 and Table 3).

### 3.4. Partially Diagnosed Patients

In 11 patients we detected one or several pathogenic variants in the heterozygous state in genes with an AR inheritance pattern. In seven cases we identified a pathogenic variant in only one gene: *USH2A* (2), *GJB2* (2), *STRC* (1), *OTOF* (1) and *CDH23* (1). In four patients we detected pathogenic variants in several different genes (Table 4)

## 4. Discussion

The genetic diagnosis of hereditary hearing loss is highly difficult due to its enormous underlying genetic heterogeneity (more than 120 genes described up to date), which is a reflection of the high complexity of the ear structure and organization. 

In the last 10 years (from 2006 to 2016), the genetic analysis of patients with hearing loss in our tertiary hospital was restricted to detect the most frequent pathogenic variants responsible for hereditary sensorineural hearing loss in Spain, specifically the complete coding sequence of the *GJB2* gene, the deletions D13S1830 and delD13S1854 in the *GJB6* gene and the *OTOF* p.Q829X variants. The implementation of our custom NGS panel containing 59 HL genes improved the management of our patients, as it has allowed us to detect putative pathogenic variants in 26 different genes. Furthermore, we have been able to genetically diagnose syndromic cases suffering from Deafness Infertility syndrome, Usher syndrome, Stickler syndrome, Waardenburg syndrome and BOR syndrome.

However, pathogenic variants in a few genes still explain a great number of hearing loss cases. The main example is *GJB2*, encoding connexin 26. Pathogenic variants in this gene are the most common cause of hereditary hearing loss in many populations [63]. In the present work, biallelic variants in *GJB2*, together with *GJB6* (DFNB1 locus), were responsible for the disease in nine families with AR inheritance, followed by pathogenic variants in *STRC* (three AR families). Regarding AD inheritance families, heterozygous pathogenic variants in *MYO6* were found in six families, followed by pathogenic variants in *TECTA* (four AD families). An additional patient presented a homozygous AR pathogenic variant in *TECTA.* All inheritance patterns have been described for HL: recessive, dominant, X-linked and mitochondrial. In some genes (like *MYO6*, *TECTA* or *ESPN*), a group of variants follow a dominant inheritance pattern, whereas others follow a recessive inheritance pattern, complicating the interpretation of genetic analysis [64]. Another feature that complicates the genetic studies of HL is the existence of some pseudogenes with high homology to some prevalent genes (like *STRC*, *OTOA* or *ESPN*). In the panel design, we tried to include some extra probes for the regions of these genes showing high homology with their pseudogenes, in addition to the default probes generated by SureDesign. However, low coverage was still obtained, and those point variants suspected to be pathogenic had to be confirmed by Sanger sequencing using primers specifically designed to hybridize only with the gene, not the pseudogene [65,66].

When a CNV affecting *STRC* or *OTOA* was suspected after DECoN v1.0.2 analysis, its presence was confirmed by MLPA using SALSA P461 (MRC Holland).

Several pathogenic variants identified in this study are reported in a large number of studies, suggesting a high prevalence. The pathogenic variant in *OTOF* c.2485C > T/p.(Gln829 *) is the third most frequent in the Spanish population that causes prelingual hearing loss [67], and *STRC* deletions are the second most frequent cause of mild-to-moderate hearing loss after the DFNB1 locus [68]. The variant c.1540C > A/p.(Gln514Lys) is the most frequent variant in *SLC26A4* in the Spanish population, described in more than 36 Spanish families to date [69]. Furthermore, the pathogenic change c.9799T > C/p.(Cys3267Arg) in *USH2A* is one of the most frequent variants in the Spanish population, specifically the third most common cause of Usher syndrome [70,71]. Finally, the pathogenic variant c.5668C T/p.(Arg1890Cys) that affects the *TECTA* gene has been described in some families from Spain, America and The Netherlands. In the most unrelated families, patients present the same haplotype, which suggests that the variant is derived from a common ancestor (founder effect) [46].

Nowadays, all known HL genes can be simultaneously analyzed thanks to the technological development of NGS. Even so, the rate of genetic diagnosis using NGS in patients with hearing loss varies around 40–60% [8,29,64,72,73,74,75,76] depending on many factors: the degree of HL (profound, severe, moderate), age of HL onset, the existence of family history, the ethnic origin or the number of genes contained in the NGS panel. The highest rates have usually been obtained for patients with a positive family history or when the HL was congenital and symmetric [8]. In the present work, the global diagnostic yield was 40%. This is a satisfactory yield, since our custom NGS panel included a limited number of genes (59), and the exclusion criteria for the genetic testing was very lax. Thus, the analyzed patient sample was very heterogeneous, including all types of sensorineural/mixed hearing loss (congenital, prelingual and postlingual; mild, moderate, severe and profound; and stable and progressive) with ages ranging from 0 to 61 years.

### 4.1. Novel VUS/Likely Pathogenic Variants

The development of NGS has revolutionized the field of genetic diagnosis, especially in extremely genetically heterogeneous diseases, such as hereditary HL. However, an elevated number of genetic variants of uncertain clinical significance (VUS) has been detected using this technology [77]. Variants predicted to generate direct stop codons or changes in the reading frame of the proteins and variants located at canonical splice sites (+/–1 and +/–2 positions of introns) are usually classified as pathological for proteins for which loss of function is reported as cause of the disease. However, the interpretation of missense, isocoding and intronic variants located out of canonical splice sites is more complex, and many times these variants remain classified as VUS. In these cases, bioinformatics predictions, segregation analyses or functional studies are required to infer the pathological character of these variants. In our study, a lot of a priori VUS variants were detected, and only seven of them were classified as likely pathogenic based upon bioinformatics predictions and/or segregation analyses: four missense, one intronic variant and one in-frame duplication. 

Missense variants: The c.235T > C/p.(Cys79Arg) change in *TMPRSS3* was not found in gnomAD exomes/genomes, and 11 computational programs predicted it as pathogenic in Varsome. Furthermore, it was found in *trans* with other previously described pathogenic variants in the *TMPRSS3* (see Table 2 and Figure 2). The *MYO6* (c.494T > G/p.(Leu165Arg)) variant was found in patient 41268. He was referred to as AD non-syndromic hearing loss, being her mother, her sister and her sister´s son were also affected. Although this variant was classified as VUS following the ACMG guidelines, we should not rule it out since it was not found in healthy control databases, had a high conservation score and showed a pathogenic computational verdict based on 13 pathogenic predictions. The *COL11A*2 (c.1748G > A/p.(Gly583Asp)) change was found in a patient and his affected father (family 37). This variant was not present in healthy population databases, and it showed pathogenic predictions in the Alissa Interpret program based on *MutationTaster*, *MutationAssessor*, *LRT*, *PolyPhen2* and *PROVEAN*. Finally, the c.977T > C/p.(Phe326Ser) (*POU3F4*) variant was found in a boy with mixed hearing loss and cochlear malformations (bilateral corkscrew cochlea, incomplete splitting of turns, absence of meatus and stapes fixation); clinical characteristics of hearing loss are linked to this gene. Furthermore, this change was absent in healthy controls databases, and it showed a pathogenic computational verdict based on 10 pathogenic predictions in Varsome.

Intronic variant: The c.1224-9del variant in *MYO6* was found in a patient with an AD pattern of inheritance in her family, given that her mother was also affected. Unfortunately, the patient´s mother refused to collaborate in the genetic study. This variant was not found in healthy control population databases, and the *MaxEnt* bioinformatic tool predicted the loss of the wild-type acceptor site. This variant was classified as VUS following the AMCG, but we consider that *MYO6* c.1224-9del could be a good candidate, and functional studies at the RNA level would be necessary to definitively confirm or discard the pathologic effect of this novel variant.

In frame duplication: The *WFS1* in-frame duplication (c.1463_1474dup/p.(Val491_Pro492insLeuIleThrVal)) was detected in a patient presenting HL also in a cousin and her son, but they did not collaborate in the study. This change was classified as VUS according to the ACMG. However, we consider that it is necessary to take this variant into account since it is not described in the population databases, has an acceptable value of conservation and, following the criteria of the ACMG, if it had been possible to show that the variant segregates correctly within the family, the WFS1 c.1463_1474dup/p.(Val491_Pro492insLeuIleThrVal) variant would be directly classified as likely pathogenic. Additionally, the clinical phenotype of this patient is similar to other patients with pathogenic variants in *WFS1*, showing a characteristic audiogram with low frequencies more affected (Figure 3). 

### 4.2. Patients with Pathogenic Variants in Two Different Genes

NGS panels allow the simultaneous analysis of a great number of genes, and, sometimes, pathogenic variants in different genes are found in the same patient. 

In the present work, the 37439 patient presented the AR c.101T > C/p.(Met34Thr) variant in *GJB2* in addition to the homozygous *STRC* whole gene deletion. Patient 39949 presented the AR c.5648G > A/p.(Arg1883Gln) variant in *MYO7A* in addition to the homozygous c.4055G > A/p.(Cys1352Tyr) AR variant in *TECTA*. These findings have important implications for reproductive genetic counseling. 

The 36163 patient was found to carry two different heterozygous novel pathogenic variants in two different genes: c.1674 + 1G > A in *MYO6* and c.2467C > T/p.(Gln823 *) in *ESPN*. Segregation analysis in this family showed that the affected father also carried the variant in *MYO*6, whereas the healthy mother carried the variant in *ESPN*. From these results it can be deduced that the variant in *MYO6* is responsible for AD hearing loss, whereas the *ESPN* variant presents an AR inheritance pattern (Figure 2). 

The 29272 and the 41950 patients from the same family carried two different previously described AD pathogenic variants in two different genes: c.2751dup/p.(Gln918Thrfs *24) in *MYO6* and c.2230G > A/p.(Asp744Asn) in *ESPN*. These two patients belong to a large family with more affected members, but these were geographically dispersed, and it was not possible to segregate these two variants with all family members in order to definitely elucidate the genetic basis and the inheritance pattern of HL in this case (Figure 2). 

Finally, the likely pathogenic novel *MYO6* (c.494T > G/p.(Leu165Arg)) variant was found in patient 41268, referred to as AD non-syndromic hearing loss. Furthermore, two previously described AR pathogenic variants in *MYO7A* (c.1997G > A/p.(Arg666Gln) and c.3527G > A/p.(Ser1176Asn)) were found in this patient. Segregation analysis would be necessary to definitely elucidate the genetic basis and the inheritance pattern of HL in this family and to offer accurate genetic reproductive genetic counseling.

### 4.3. Syndromic Cases

Most patients included in this study suffered from non-syndromic hearing loss, but eight cases were referred as syndromic: four patients with Usher syndrome (USH), two Waardenburg syndrome (WS) patients and two branchio-oto-renal syndrome (BOR) patients. We could find the variants responsible for the disease in five of them (Table 2A,B). 

The patient 35238 and his mother (42783) were referred as NSHL, but they were found to carry a pathogenic variant in *COL11A2*: c.4392 + 1G > A. This variant had been previously reported by Brunner et al. (1994) [48] associated with Stickler syndrome without eye affectation. These patients were clinically re-evaluated, and both presented with osteoarticular problems and flattened facial profiles (Table 2B). Thus, this family was re-classified as Stickler syndrome. 

Three unrelated cases with bilateral, symmetrical, postlingual, moderate and stable HL (33416, 37112 and 37439) presented biallelic contiguous-gene deletions at chromosome 15q15.3 that included both *CATSPER2* and *STRC.* This deletion causes deafness–infertility syndrome (DIS) in males due to *CATSPER* haploinsufficiency results in sperm abnormalities [78]. The patient 37112 was a male of 4 years old. Thus, the patient´s parents were informed that their son will be infertile in adulthood. 

**Figure 4:**
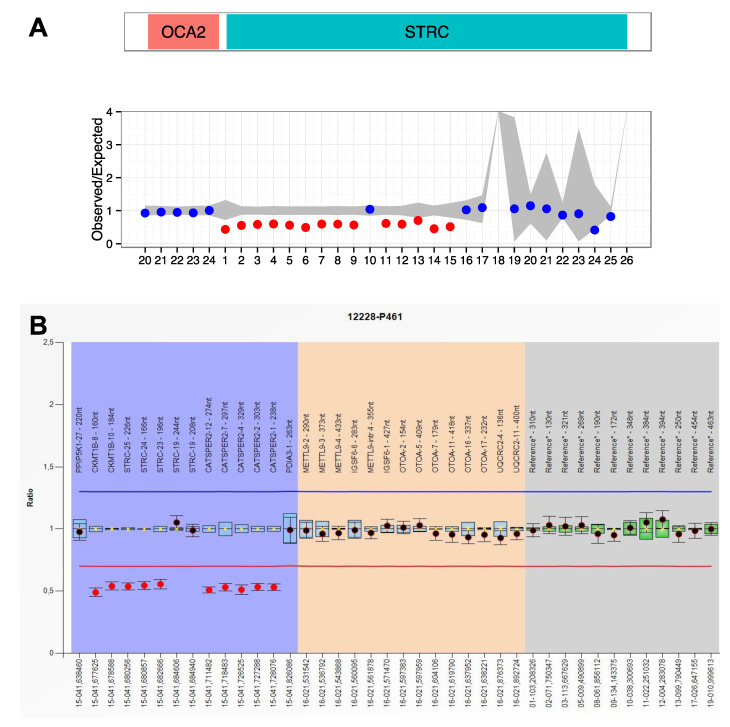
Complex rearrangement identified in patient 12228 in the *STRC* gene. (**A**) Result obtained from *Decon* software. The x-axis represents the exon number. Blue points reflect a normal value. Red points reflect a possible deletion for the exon. (**B**) MLPA representation of patient 12228 with the P461 salsa using the Coffalyzer.Net program (MRC Holland). Normal range: 0.7–1.3 (indicated with red and blue line, respectively).

In another case (12228), a complex rearrangement involving *STRC* and *CATSPER2* was detected (Table 3 and Figure 4). *DECoN* analysis using NGS data showed a partial deletion involving exons 1–15 of *STRC*. However, based on coverage data from NGS, we could not differentiate between *STRC* and *pSTRC*. Thus, an MLPA analysis was performed using P-461 SALSA (MRC Holland). This SALSA includes specific probes only for exons 19, 24–25 of *STRC* and also some specific probes for some exons of *CATSPER2*. MLPA results showed a partial deletion affecting exons 23, 24 and 25 of *STRC* (chr15:41680256-41682666), whereas *STRC* exon 19 showed a normal dosage (chr15:41684606-41684940). However, chromosome coordinates chr15:41711482-41728076 corresponding to *CATSPER2* showed again a ratio of 0.5. Segregation analysis would be helpful in this case to find out if this complex rearrangement is carried in the same chromosome or if there are two different deletions affecting the 15q15.3 locus, located in different alleles.

## 5. Conclusions

A large number of genes has been associated with HL, but still many cases remain unexplained. Novel HL genes are expected to be discovered and also genetic variants affecting regulatory regions of the genome, which are currently not screened in diagnosis genetic testing. Furthermore, the possibility of multigenic inheritance patterns must be explored in the near future [79]. 

Nowadays, a huge number of DNA variants are being detected in countless genetic diagnostic laboratories around the world, and a non-negligible number of them are possibly being misinterpreted. It is necessary to share this information with the scientific community and to establish close collaborations to interpret the functional implications of DNA variability. Working altogether, we will be able to decipher the secrets that we still ignore about the human genome. 

## Figures and Tables

**Table 1:** The Table Indicates the Genes Included in this Study and the Associated Phenotype.

Gene	Phenotype	Gene	Phenotype
*ACTG1*	NSHL	*TRIOBP*	NSHL
*CEP250*	NSHL	*CDH23*	USH/NSHL
*CHD7*	CHARGE	*CIB2*	USH/NSHL
*CISD2*	NSHL	*DFNB31*	USH/NSHL
*CLDN14*	NSHL	*MYO7A*	USH/NSHL
*COCH*	NSHL	*PCDH15*	USH/NSHL
*DFNA5*	NSHL	*USH1C*	USH/NSHL
*DFNB59*	NSHL	*USH1G*	USH/NSHL
*ESPN*	NSHL	*EDN3*	WS
*EYA4*	NSHL	*EDNRB*	WS
*GJB2*	NSHL	*MITF*	WS
*GJB6*	NSHL	*PAX3*	WS
*KCNQ4*	NSHL	*SNAI2*	WS
*LHFPL5*	NSHL	*SOX10*	WS
*LOXHD1*	NSHL	*EYA1*	BOR
*LRTOMT*	NSHL	*SIX1*	BOR
*MYH9*	NSHL	*SIX5*	BOR
*MYH14*	NSHL	*ADGRV1*	USH
*MYO6*	NSHL	*CLRN1*	USH
*MYO15A*	NSHL	*USH2A*	USH
*OTOA*	NSHL	*KCNE1*	JLNS
*OTOF*	NSHL	*KCNQ1*	JLNS
*OTOG*	NSHL	*COL11A2*	Stickler/NSHL
*OTOGL*	NSHL	*SEMA3E*	CHARGE
*POU3F4*	NSHL	*SLC26A4*	Pendred/NSHL
*PTPRQ*	NSHL	*WFS1*	WF/NSHL
*SMPX*	NSHL	chr1:215827262-215827362	USH
*STRC*	NSHL	chr1:215967733-215967833	USH
*TECTA*	NSHL	chr1:216039671-216039771	USH
*TIMM8A*	NSHL	chr1:216064520-216064560	USH
*TMC1*	NSHL	chr1:216247426-216247526	USH
*TMPRSS3*	NSHL		
*TPRN*	NSHL		

NSHL: Non-syndromic hearing loss, USH: Usher syndrome, WS: Waardenburg syndrome, BOR: BOR syndrome, JLNS: Jervell and Lange-Nielsen syndrome, Stickler: Stickler syndrome, CHARGE: Charge syndrome, Pendred: Pendred syndrome, WF: Wolfram syndrome.

**Table 2:** Disease Causing Variants Detected and Clinical Data of the Diagnosed Patients.

(A) Patients Diagnosed with Autosomal Recessive Deafness												
Family	Patient		Sex		Age	Diagnosis		Gene		Allele 1	Allele 2	Phenotype
1	33311		M		1	NSHL		*GJB2* NM_004004.5		c.35del/p.(Gly12Valfs *2) [16]	c.35del/p.(Gly12Valfs *2) [16]	SNHL, bilateral, symmetrical, prelingual, severe, stable
2	35961		F		2	NSHL		*GJB2* NM_004004.5		c.35del/p.(Gly12Valfs *2) [16]	c.35del/p.(Gly12Valfs *2) [16]	SNHL, bilateral, symmetrical, prelingual, moderate, stable
3	39026		F		0	NSHL		*GJB2* NM_004004.5		c.35del/p.(Gly12Valfs *2) [16]	c.35del/p.(Gly12Valfs *2) [16]	SNHL, bilateral, symmetrical, prelingual, severe-profound, stable
4	39611		F		5	NSHL		*GJB2* NM_004004.5		c.596C > T/p.(Ser199Phe) [17]	c.35del/p.(Gly12Valfs *2) [16]	SNHL, bilateral, symmetrical, postlingual, severe, stable
5	40372		M		5	NSHL		*GJB2* NM_004004.5		c.617A > G/p.(Asn206Ser) [18]	c.269dup/p.(Val91Serfs *11) [19]	SNHL, bilateral, symmetrical, postlingual, mild–moderate, stable
6	42105		M		0	NSHL		*GJB2* NM_004004.5		c.101T > C/p.(Met34Thr) [20]	c.427C > T/p.(Arg143Trp) [21]	SNHL, bilateral, symmetrical, prelingual, moderate, stable
7	28981		M		0	NSHL		*GJB2* NM_004004.5		c.35del/p.(Gly12Valfs *2) [16]		SNHL, bilateral, symmetrical, prelingual, profound, stable
								*GJB6* NM_001110219.2			del(GJB6-D13S1830) [22]	
8	34307		M		0	NSHL		*GJB2* NM_004004.5		c.269dup/p.(Val91Serfs *11) [19]		SNHL, bilateral, symmetrical, prelingual, profound, stable
								*GJB6* NM_001110219.2			del(GJB6-D13S1830) [22]	
9	37468		M		1	NSHL		*GJB2* NM_004004.5		c.617A > G/p.(Asn206Ser) [18]		SNHL, bilateral, symmetrical, prelingual, profound, stable
								*GJB6* NM_001110219.2			del(GJB6-D13S1830) [22]	
10	37439		F		5	NSHL		*STRC* NM_153700.2		Whole gene deletion (15q15) [23]	Whole gene deletion (15q15) [23]	SNHL, bilateral, symmetrical, postlingual, moderate, stable
								*GJB2* NM_004004.5		c.101T > C/p.(Met34Thr) [20]		
11	33416		F		7	NSHL		*STRC* NM_153700.2		Whole gene deletion (15q15) [23]	Whole gene deletion(15q15) [23]	SNHL, bilateral, symmetrical, postlingual, moderate, stable
12	37112		M		4	NSHL		*STRC* NM_153700.2		Whole gene deletion (15q15) [23]	Whole gene deletion (15q15) [23]	SNHL, bilateral, symmetrical, postlingual, moderate, stable
13	31410		M		5	NSHL		*OTOF* NM_194248.2		c.4275G > A/p.(Trp1425 *) [24]	c.2485C > T/p.(Gln829 *) [25]	SNHL, bilateral, symmetrical, prelingual, profound, stable
14	40184		F		0	NSHL		*OTOF* NM_194248.2		c.2485C > T/p.(Gln829 *) [25]	c.2485C > T/p.(Gln829 *) [25]	SNHL, bilateral, symmetrical, prelingual, profound, stable
14	41793		F		0	NSHL		*OTOF* NM_194248.2		c.2485C > T/p.(Gln829 *) [25]	c.2485C > T/p.(Gln829 *) [25]	SNHL, bilateral, symmetrical, prelingual, profound, stable
15	34197		F		54	NSHL		*LOXHD1* NM_144612.6		c.3419dup/p.(Leu1140Phefs *5)	c.3419dup/p.(Leu1140Phefs *5)	SNHL, bilateral, symmetrical, postlingual, moderate–severe, stable
16	34865		M		7	NSHL		*LOXHD1* NM_144612.6		c.4480C > T/p.(Arg1494 *) [26]	c.4480C > T/p.(Arg1494 *) [26]	SNHL, bilateral, symmetrical, postlingual, moderate, stable
17	29440		M		33	NSHL		*OTOA* NM_144672.3		c.877C > T/p.(Gln293 *)	Whole gene deletion (16q12.2 region) [27]	SNHL, bilateral, postlingual, moderate, stable
18	37140		M		4	NSHL		*OTOA* NM_144672.3		Whole gene deletion (16q12.2 region) [27]	Whole gene deletion (16q12.2 region) [27]	SNHL, bilateral, symmetrical, postlingual, moderate, stable
19	29865		F		46	NSHL		*TMPRSS3* NM_024022.2		c.1276G > A/p.(Ala426Thr) [28]	c.1159G > A/p.(Ala387Thr) [29]	SNHL, bilateral, symmetrical, postlingual, mild–moderate, progressive
19	38198		F		40	NSHL		*TMPRSS3* NM_024022.2		c.1276G > A/p.(Ala426Thr) [28]	**c.235T > C/p.(Cys79Arg)**	SNHL, bilateral, symmetrical, postlingual, profound, progressive
20	42108		F		1	NSHL		*MYO15A* NM_016239.3		c.8968-1G > T [30]	c.8968-1G > T [30]	SNHL, bilateral, symmetrical, prelingual, severe, stable
21	37513		M		4	NSHL/EVA		*SLC26A4* NM_000441.1		c.1540C > A/p.(Gln514Lys) [31]	c.1540C > A/p.(Gln514Lys) [31]	SNHL, bilateral, asymmetrical, postlingual, Right: profound Left: moderate, stable, EVA
22	36777		F		1	NSHL		*OTOG* NM_001277269.1		c.2140dup/p.(Ser714Lysfs *22)	c.2140dup/p.(Ser714Lysfs *22)	SNHL, bilateral, symmetrical, prelingual, moderate, stable
23	39949		F		18	NSHL		*TECTA* NM_005422.2		c.4055G > A/p.(Cys1352Tyr) [32]	c.4055G > A/p.(Cys1352Tyr) [32]	SNHL, bilateral, moderate
								*MYO7A* NM_000260.3		c.5648G > A/p.(Arg1883Gln) [33]		
24	40453		F		40	NSHL		*MYO7A* NM_000260.3		c.1232T > C/p.(Val411Ala) [34]	c.6025del/p.(Ala2009Profs *32) [35]	SNHL, bilateral, symmetrical, postlingual, mild, stable
25	27862		M		30	USH		*ADGRV1* NM_032119.3		c.12528-1G > T [36]	c.17933A > G/p.(His5978Arg) [37]	SNHL, congenital, moderate, retinitis pigmentosa
26	30816		F		1	USH		*CDH23* NM_022124.5		c.310G > T/p.(Glu104*)	c.2289 + 1G > A [38]	SNHL, bilateral, symmetrical, prelingual, profound, stable, bilateral, vestibular areflexia, retinitis pigmentosa
27	27734		F		49	USH		*USH2A*NM_206933.2		c.9799T > C/p.(Cys3267Arg) [39]	c.9676C > T/p.(Arg3226 *) [40]	SNHL, bilateral, symmetrical, postlingual, moderate, stable, retinitis pigmentosa
(B) Patients Diagnosed with Autosomal Dominant Deafness												
Family	Patient		Sex		Age	Diagnosis		Gene		Allele 1	Allele 2	Phenotype
28	32954		M		46	NSHL		*MYO6* NM_004999.3		c.2545C > T/p.(Arg849 *) [41]		SNHL, bilateral, symmetrical, postlingual, moderate, stable
28	32955		F		15	NSHL		*MYO6* NM_004999.3		c.2545C > T/p.(Arg849 *) [41]		SNHL, bilateral, symmetrical, moderate, stable
29	35197		F		37	NSHL		*MYO6* NM_004999.3		c.1666C > T/p.(Arg556 *)		SNHL, bilateral, symmetrical, postlingual, moderate, stable
30	40488		F		30	NSHL		*MYO6* NM_004999.3		**c.1224-9del**		SNHL, bilateral, asymmetrical, postlingual, severe–profound, progressive
31	31110		M		42	NSHL		*MYO6* NM_004999.3		**c.1674 + 1G > A**		SNHL, bilateral, symmetrical, postlingual, moderate, stable
31	36163		M		32	NSHL		*MYO6* NM_004999.3		**c.1674 + 1G > A**		SNHL, bilateral, symmetrical, postlingual, moderate, stable
								*ESPN* NM_031475.2		c.2467C > T/p.(Gln823 *)		
32	29272		M		46	NSHL		*MYO6* NM_004999.3		c.2751dup/p.(Gln918Thrfs *24)[42]		SNHL, postlingual
								*ESPN* NM_031475.2		c.2230G > A/p.(Asp744Asn) [43]		
32	41950		F		61	NSHL		*MYO6* NM_004999.3		c.2751dup/p.(Gln918Thrfs *24) [42]		SNHL, postlingual
								*ESPN* NM_031475.2		c.2230G > A/p.(Asp744Asn) [43]		
33	41268		M		18	NSHL		*MYO6* NM_004999.3		**c.494T > G/p.(Leu165Arg)**		SNHL, bilateral, symmetrical, postlingual, profound, progressive, tinnitus
								*MYO7A* NM_000260.3		c.1997G > A/p.(Arg666Gln) [44]	c.3527G > A/p.(Ser1176Asn) [8]	
34	33945		M		3	NSHL		*TECTA* NM_005422.2		c.5668C > T/p.(Arg1890Cys) [45]		SNHL, bilateral, asymmetrical prelingual, stable
35	35453		M		2	NSHL		*TECTA* NM_005422.2		c.5383 + 5_5383 + 8del [46]		SNHL, bilateral, symmetrical, prelingual, moderate, stable
36	38971		F		0	NSHL		*TECTA* NM_005422.2		c.5509T > G/p.(Cys1837Gly) [47]		SNHL, bilateral, symmetrical, prelingual, moderate, stable
36	39927		F		0	NSHL		*TECTA* NM_005422.2		c.5509T > G/p.(Cys1837Gly)[47]		SNHL, unilateral, asymmetrical, prelingual, moderate–severe, progressive
37	4293		M		6	NSHL		*COL11A2* NM_080680.2		**c.1748G > A/p.(Gly583Asp)**		SNHL, bilateral, symmetrical, postlingual, stable
37	31449		M		35	NSHL		*COL11A2* NM_080680.2		**c.1748G > A/p.(Gly583Asp)**		SNHL, bilateral, asymmetrical, postlingual, Right: mild–moderate; Left: moderate–severe, stable
38	35238		M		6	NSHL/Stickler		*COL11A2* NM_080680.2		c.4392 + 1G > A [48]		SNHL, bilateral, symmetrical, postlingual, moderate, stable, flattened facial profile, sunken nasal root, short nose with anteverted nostrils, osteorticular problems
38	42783		F		37	NSHL/Stickler		*COL11A2* NM_080680.2		c.4392 + 1G > A [48]		SNHL, flattened facial profile, osteorticular problems and maxillofacial alterations
39	40431		M		5	NSHL		*WFS1* NM_006005.3		**c.1463_1474dup/p.(Val491_Pro492insLeuIleThrVal)**		SNHL, bilateral, asymmetrical, postlingual, Right: profound Left: severe, progressive
40	42125		F		5	NSHL		*WFS1* NM_006005.3		c.2108G > A/p.(Arg703His) [49]		SNHL, bilateral, symmetrical, postlingual, severe–profound, progressive
41	36655		M		7	NSHL		*KCNQ4* NM_004700.3		c.857A > G/p.(Tyr286Cys) [50]		SNHL, bilateral, symmetrical, postlingual, moderate, stable
41	44138		M		46	NSHL		*KCNQ4* NM_004700.3		c.857A > G/p.(Tyr286Cys) [50]		
42	39490		F		45	NSHL		*ACTG1* NM_001199954.1		c.895C > G/p.(Leu299Val) [29]		SNHL, bilateral, asymmetrical, postlingual, right: moderate left: severe, progressive
43	40519		M		40	NSHL		*EYA4* NM_004100.4		c.988C > T/p.(Gln330 *) [51]		SNHL, bilateral, asymmetrical, postlingual, right: profound left: severe, progressive, tinnitus, decrease in size of both cochlear nerves
44	12227		M		34	WS		*MITF* NM_198159.2		c.943C > T/p.(Arg315 *) [52]		HL, prelingual, White forelock, Heterochromia iridis
								*GJB6* NM_001110219.2		del(GJB6-D13S1830) [22]		
45	37350		M		2	BOR		*EYA1* NM_000503.5		c.1540_1542del/p.(Leu514del) [53]		Mixed HL, bilateral, symmetrical, prelingual, severe, stable, 2nd branchial arch fistula, facial dysmorphia
**(C) Patients Diagnosed with X-Linked Deafness**												
**Family**	**Patient**	**Sex**		**Age**		**Diagnosis**	**Gene**		**Allele 1**		**Allele 2**	**Phenotype**
46	34796	M		1		NSHL	*POU3F4* (XLR) NM_000307.4		**c.977T > C/p.(Phe326Ser)**			Mixed HL, bilateral symmetrical, prelingual, moderate, stable, bilateral corkscrew cochlea, incomplete splitting of turns, absence of meatus and stapes fixation
47	14285	M		3		NSHL	*SMPX* (XLD) NM_014332.2		c.20del/p.(Pro7Glnfs *74)			SNHL, bilateral, symmetrical, postlingual, moderate, stable
47	41863	F		30		NSHL	*SMPX* (XLD) NM_014332.2		c.20del/p.(Pro7Glnfs *74)			SNHL, bilateral, symmetrical, postlingual, moderate, stable

The table indicates the patient and family code, sex, age (indicated in years), diagnosis, mutated gene, variants and phenotype. The variants described in the table are pathogenic or probably pathogenic, and novel variants are marked in bold. M: male, F: female, NSHL: non-syndromic hearing loss, NHL: sensorineural hearing loss, HL: hearing loss, EVA: enlarged vestibular aqueduct, USH: Usher syndrome, WS: Waardenburg syndrome, BOR: branchio–oto–renal, XLR: recessive X-linked, XLD: dominant X-linked.

**Table 3:** Classification of Novel Variants Identified in this Study.

	Variant			Frequency			Pathogenicity Scores		
Gene	Nucleotide	Protein	Classification	GnomAD Exomes	GnomAD Genomes	Deafness Variation Database	Missense Pathogenicity Scores	Conservation Score (GERP)	MaxEnt
*LOXHD1* NM_144612.6	c.3419dup	p.(Leu1140Phefs *5)	Pathogenic	0.0000267	NF	NF	NA	5.05	-
*OTOA* NM_144672.3	c.877C > T	p.(Gln293 *)	Pathogenic	NF	NF	NF	NA	5.41	-
*TMPRSS3* NM_024022.2	c.235T > C	p.(Cys79Arg)	Likely Pathogenic	NF	NF	NF	11/13	5.23	-
*OTOG* NM_001277269.1	c.2140dup	p.(Ser714Lysfs *22)	Pathogenic	NF	NF	NF	NA	4.9	-
*CDH23* NM_022124.5	c.310G > T	p.(Glu104 *)	Pathogenic	NF	NF	NF	NA	5.43	-
*MYO6* NM_004999.3	c.1666C > T	p.(Arg556 *)	Pathogenic	0.0000119	NF	Unknown significance–Impact High	NA	5.77	-
*MYO6* NM_004999.3	c.1224-9del	-	VUS	NF	NF	NF	NA	5.23	AS broken (from 7.08 to −4.37)
*MYO6* NM_004999.3	c.1674 + 1G > A	-	Pathogenic	0.00000736	NF	Unknown significance-Impact High	NA	5.77	DS broken (from 7.94 to −0.24)
*ESPN* NM_031475.2	c.2467C > T	p.(Gln823 *)	Pathogenic	NF	NF	NF	NA	4.28	-
*MYO6* NM_004999.3	c.494T > G	p.(Leu165Arg)	VUS	NF	NF	NF	13/13	5.45	-
*COL11A2* NM_080680.2	c.1748G > A	p.(Gly583Asp)	Likely Pathogenic	NF	NF	NF	11/11	3.89	-
*WFS1* NM_006005.3	c.1463_1474dup	p.(Val491_Pro492insLeuIleThrVal)	VUS	NF	NF	NF	NA	4.25	-
*POU3F4* NM_000307.4	c.977T > C	p.(Phe326Ser)	Likely Pathogenic	NF	NF	NF	10/10	5.07	-
*SMPX* NM_014332.2	c.20del	p.(Pro7Glnfs *74)	Pathogenic	NF	NF	NF	NA	5.78	-

NF: not found; NA: not available; AS: acceptor splice-site; DS: donor splice-site. “Classification”: Variants are classified according to the guidelines of the ACMG [54].“Pathogenicity Scores” refer to the number of in silico tools that classify the variant as pathogenic/likely pathogenic versus the total of predictors used. The scores were obtained from https://Varsome.com/ (accessed November 2020) and included the followings predictors: *BayesDel_addAF, DANN, DEOGEN2, EIGEN,FATHMM-MKL, LIST-S2, M-CAP, MVP, MutationAssessor, MutationTaster, PrimateAI, REVEL* and *SIFT*. Not all predictors were available for all analyzed variants. GERP is a conservation score. The values range from −12.3 to 6.17, with 6.17 being the most conserved.

**Table 4:** Patients with only One Heterozygous Pathogenic or Likely Pathogenic Variant in Genes Associated with an Autosomal Recessive Inheritance Pattern.

Patient	Diagnosis	Gene	Allele 1
40056	NSHL	*USH2A* NM_206933.2	c.4325T > C/p.(Phe1442Ser) [55]
31443	USH	*USH2A* NM_206933.2	c.2431_2432del/p.(Lys811Aspfs*11) [35]
28523	NSHL	*USH2A* NM_206933.2	c.2135del/p.(Ser712*) [56]
		*MYO7A* NM_000260.3	c.5581C > T/p.(Arg1861*) [57]
37248	NSHL	*USH2A* NM_206933.2	c.9244A > G/p.(Ile3082Val) [58]
		*GJB2* NM_004004.5	c.109G > A/p.(Val37Ile) [59]
37986	NSHL	*GJB2* NM_004004.5	c.269T > C/(p.Leu90Pro) [19]
39353	NSHL	*GJB2* NM_004004.5	c.445G > A/p.(Ala149Thr) [60]
12228	NSHL	*STRC* NM_153700.2	**Complex rearrangement**
28358	NSHL	*OTOF* NM_194248.2	c.2485C > T/p.(Gln829*) [25]
35862	NSHL	*OTOF* NM_194248.2	c.2485C > T/p.(Gln829*) [25]
		*CDH23* NM_022124.5	c.4762C > T/p.(Arg1588Trp) [61]
33335	USH	*CDH23* NM_022124.5	c.2289 + 1G > A [38]
34978	NSHL	*TMC1* NM_138691.2	c.1763 + 3A > G [62]
		*TMPRSS3* NM_024022.2	c.280G > A/p.(Gly94Arg) [29]

NSHL: non-syndromic hearing loss, USH: Usher syndrome. Novel variants are marked in bold.

## Supplementary Materials

The following are available online at https://www.mdpi.com/2073-4425/11/12/1467/s1, Table S1: Regions of the panel design with a poor coverage.
