# Supplementary material for: Improving the Management of Patients with Hearing Loss by the Implementation of an NGS Panel in Clinical Practice
Source: Genes (Basel). 2020 Dec 7;11(12):1467. doi: 10.3390/genes11121467 (PMC7762334; doi:10.3390/genes11121467)
Supplement: Supplementary file 1 [file genes-11-01467-s001.pdf]

Table S1. Regions of the Panel Design with A Poor Coverage.

	Region		Gene	NM	Exon	% of the Region not Covered
	Start	End				
chr1	6484847	6485309	*ESPN*	NM_031475	1	5%
chr1	6500313	6500500	*ESPN*	NM_031475	3	50%
chr1	6500685	6500868	*ESPN*	NM_031475	4	40%
chr1	6505723	6505995	*ESPN*	NM_031475	7	75%
chr1	6508700	6509151	*ESPN*	NM_031475	8	12%
chr1	6511662	6511808	*ESPN*	NM_031475	9	13%
chr16	21742157	21742251	*OTOA*	NM_144672	20	90%
chr16	21747581	21747711	*OTOA*	NM_144672	21	20%
chr16	21752041	21752229	*OTOA*	NM_144672	22	100%
chr16	21756201	21756357	*OTOA*	NM_144672	23	100%
chr16	21763255	21763398	*OTOA*	NM_144672	24	100%
chr16	21763689	21763826	*OTOA*	NM_144672	25	100%
chr16	21768402	21768598	*OTOA*	NM_144672	27	100%
chr16	21771790	21772050	*OTOA*	NM_144672	28	100%
chr15	43891760	43891958	*STRC*	NM_153700	29	75%
chr15	43892157	43892305	*STRC*	NM_153700	28	65%
chr15	43892731	43892880	*STRC*	NM_153700	26	8%
chr15	43893069	43893212	*STRC*	NM_153700	25	6%
chr15	43896193	43896350	*STRC*	NM_153700	22	100%
chr15	43896847	43897044	*STRC*	NM_153700	20	50%
chr15	43897461	43897597	*STRC*	NM_153700	19	25%
chr15	43900060	43900173	*STRC*	NM_153700	18	30%
chr15	43900277	43900401	*STRC*	NM_153700	17	50%
chr15	43901473	43901532	*STRC*	NM_153700	16	100%
chr15	43902509	43902635	*STRC*	NM_153700	15	90%
chr15	43903116	43903182	*STRC*	NM_153700	14	100%
chr15	43903345	43903513	*STRC*	NM_153700	13	50%
chr15	43903696	43903735	*STRC*	NM_153700	12	100%
chr15	43904039	43904222	*STRC*	NM_153700	11	100%
chr15	43904574	43904707	*STRC*	NM_153700	10	100%
chr15	43904988	43905104	*STRC*	NM_153700	9	100%
chr15	43905242	43905429	*STRC*	NM_153700	8	100%
chr15	43906084	43906251	*STRC*	NM_153700	7	100%
chr15	43906393	43906510	*STRC*	NM_153700	6	100%
chr15	43906611	43906674	*STRC*	NM_153700	5	85%
chr15	43907630	43908888	*STRC*	NM_153700	4	100%
chr15	43909655	43909680	*STRC*	NM_153700	3	100%
chr15	43909768	43910554	*STRC*	NM_153700	2	30%
chr15	43910856	43910998	*STRC*	NM_153700	1	90%
